# Functional anatomy of the sharpshooter precibarial valve supports its role in probing behaviors that control inoculation of *Xylella fastidiosa*

**DOI:** 10.1038/s41598-025-14208-4

**Published:** 2025-09-30

**Authors:** Elaine A. Backus, Damien Laudier

**Affiliations:** 1 San Joaquin Valley Agricultural Sciences Center, USDA Agricultural Research Service, 9611 South Riverbend Avenue, Parlier, 93648-9757 CA USA; 2 Laudier Histology, P.O. Box 78, New York, 10025 NY USA

**Keywords:** Insecta, Cicadellinae, Pierce’s disease, Olive quick decline, Feeding, Egestion, Structural biology, Zoology

## Abstract

The precibarial valve is a tiny structure in the functional foregut (cibarium and precibarium) of hemipteran insects. Piercing-sucking insects like hemipterans use straw-like mouth parts (stylets) to ingest fluid from plant vascular cells like phloem or xylem. Suction is provided by the cibarium (or cibarial pump), which draws fluid through the stylets into a short canal called the precibarium, then into the cibarium from which the fluid is swallowed into the pharynx then esophagus. The precibarium houses two sets of chemosensilla separated by the tiny precibarial valve, which is operated by its own muscle independent of the muscles powering the cibarium. Despite intensive study, the operational mechanism of the precibarial valve in sharpshooter leafhoppers is not known because the muscle attachment to the valve has not been clearly identified. Such an operational mechanism is important because it controls inoculation of the lethal bacterial pathogen *Xylella fastidiosa* into healthy crop plants, causing economically devastating diseases such as Pierce’s disease of grape, citrus variegated chlorosis, olive quick decline, and numerous leaf scorches. Together, these diseases cause many $billions of damage and control costs worldwide, annually. The present study provides definitive knowledge of how the precibarial valve muscle attaches to the valve in sharpshooter vectors of *Xylella fastidiosa*. It also proposes a new operation model to control fluid flows responsible for inoculating the pathogen into healthy plants. Such information will aid in development of novel disease management methods such plant resistance to vector performance of inoculation behaviors.

**Supplementary Information:**

The online version contains supplementary material available at 10.1038/s41598-025-14208-4.

## Introduction

The present study provides updated understanding of how the precibarial valve in the foreguts of sharpshooter leafhopper vectors of *Xylella fastidiosa* (*Xf*) controls feeding behaviors responsible for inoculating the pathogen into healthy plants. This is important because *Xylella fastidiosa* is an invasive, xylem-limited, bacterial plant pathogen that causes lethal scorch diseases on a wide array of economically important horticultural crops. Insect vectors (carriers) are piercing-sucking hemipteran insects specialized to ingest primarily xylem sap^1^. Sharpshooter leafhoppers (Hemiptera: Auchenorrhyncha: Cicadellidae: Cicadellinae) like the blue-green sharpshooter (*Graphocephala atropunctata* Say) and the glassy-winged sharpshooter (*Homalodisca vitripennis* Germar) are the primary vectors in California of Pierce’s disease, a lethal *Xf*-caused disease of grapes, which costs over $100 million per year in California alone to control^2^. Similarly, the spittlebug *Philaenus spumarius* L. (Auchenorrhyncha: Aphrophoridae) is the vector of recently-introduced olive quick decline in Italy, which has caused the loss of $billions in damage and control^1,3^.

Vector-borne plant pathogens like *Xf* pose a growing threat to agriculture, due to human transport of pathogens and climate change. One of the best alternatives to present use of pesticides is disease-resistant crops, although breeding resistance to vector-borne plant pathogens is challenging^4^. Meeting this challenge for *Xf* depends in part on intimately understanding how *Xf* inoculation is performed by the vector. Using electropenetrography (EPG), waveforms have been identified that represent vector behaviors responsible for inoculating *Xf* into a xylem cell^5–7^. Resistance to the vector’s ability to perform these inoculation behaviors would improve field durability of resistance^8^. Knowing the intimate details underlying *Xf* inoculation, especially operation of the precibarial valve, can aid in development of such resistance, but obstacles remain.

**Fig. 1:**
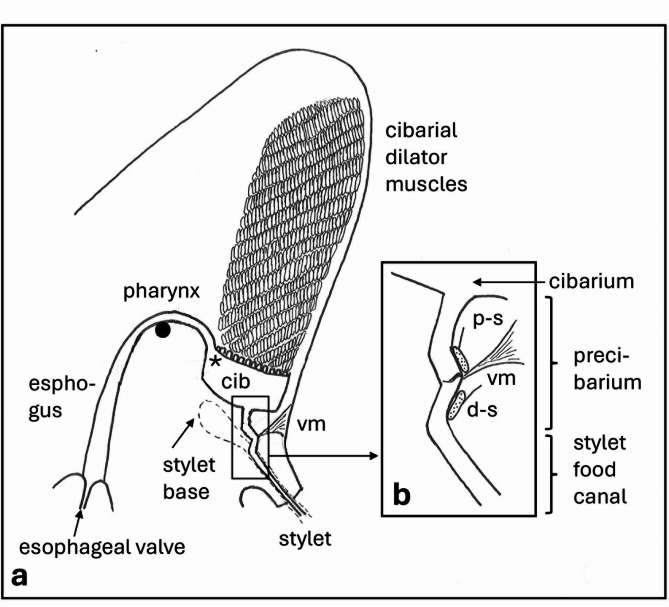
Side view of sharpshooter head. (**a**) Overview of structures described in text to show their sizes and relative positions in the functional foregut. Small box near the cibarium (cib) is enlarged in the inset. *, location of the true mouth; vm, precibarial valve muscle; large black dot, cross section of the supporting cuticular bridge (tentorium). (**b**) The fluid-conducting channel formed by the convergence of the stylet food canal, precibarium, and opening to the cibarium. d-s, neuron cell bodies and nerve (short line) from the distal chemosensilla; p-s, cell bodies and nerve from the proximal chemosensilla (drawing by E. Backus).

Uniquely, *Xf* is the only known insect-transmitted, plant pathogen that both colonizes and multiplies on the surface of the functional foregut of the vector. The functional foregut is composed of the cibarium (sucking pump) and precibarium, a narrow canal that conveys fluid from the food canal in the piercing-sucking mouth parts (stylets) to the cibarium before the food is swallowed past the true mouth (Fig. 1). *Xf* cells are expelled directly from the functional foregut into the plant during egestion (fluid flow outward from the stylet tips). Egestion is the “out” part of rapid “in-out-in-out” fluid movement the insect performs prior to subsequent sustained ingestion (swallowing); the latter is ultimately performed for hours^5^. Many years of study plus recent definitive support have shown that egestion is the chief *Xf* inoculation behavior^5,9–12^but its mechanisms are still unknown.

Unlocking the mechanisms of egestion and therefore *Xf* inoculation depend upon understanding the critical structures, and operations of the precibarium^5,11^. The anatomy of the precibarium is complex; it houses two sets of chemosensilla with a tiny valve in between that is independently moved by its own muscle (Fig. 1)^5,10,13^. Much still remains unknown about the operations of the precibarial valve. Yet, its structures and their functions are the key for understanding the mechanisms of egestion and therefore *Xf* inoculation^5,12^. While the hydrodynamics of fluid inflow and outflow through the functional foregut are known^10,11^^13^. it is not known how the valve controls those dynamics to perform two hypothesized types of egestion, (1) rinsing egestion (or dribbling) wherein small amounts of fluid are slowly expelled, and (2) discharging egestion (or spitting) wherein larger amount of fluid are rapidly expelled^5,9^. Mechanisms are discussed at length in the Discussion.

Five studies have used scanning electron microscopy (SEM) of sharpshooters to view the lumen of the functional foregut of sharpshooters when the apposed cuticular plates creating it (epipharynx and hypopharynx) are split apart^14–18^. Of these studies, only one^15^ cut open the epipharynx to view the precibarial valve muscle to attempt to discern its operational mechanism. However, the attachment of the muscle to the valve was not visible (Supplementary **Fig. S1**). No studies have been able to section through for light microscopy (LM) and view the intact interior of the precibarial valve in sharpshooters. Two other studies examined the precibarium of the spittlebug *P. spumarius*; the first used SEM and showed that the appearance of the precibarium was similar to those of sharpshooters^19^. The second sectioned through the precibarium for both LM and transmission electron microscopy (TEM) to attempt to view the attachment of the precibarial valve muscle to the valve^12^. Yet, even from that study, no clear and unambiguous image of the muscle attachment to the precibarial valve was shown. Nonetheless, the latter authors propose a complicated operational mechanism for the precibarial valve of *P. spumarius* without definitive anatomical evidence of attachment and suggest that the same mechanism also applies to sharpshooters. Their model shows indirect attachment and movement of the valve (Supplementary **Fig. S2**), with implications for fluid flow and *Xf* inoculation.

The objective of the present study was to definitively identify the muscle attachment to the precibarial valve and to derive a model for valve operation in two sharpshooters, *G. atropunctata* and *H. vitripennis*. Modern resin embedment and processing techniques were used to prevent the tiny, cuticular structures of the valve from “popping out” of polymerized resin during sectioning, a common problem for previous studies. Findings described herein definitively showed that muscle attachment to the valve in sharpshooters is not indirect^12^but direct and hinged via three complex, elasticized attachments. Also, this work confirmed that the valve muscle is relaxed when the valve is closed, as with spittlebugs^12^opposite of what was originally proposed when the valve was discovered in leafhoppers^20^. The parsimonious operational model derived from these findings supports that the precibarial valve and its muscular control regulate fluid movement during stylet probing. In addition, this work directly visualized for the first time unknown microbes (but similar in size and biofilm formation to *Xf*) suspended in the distal part of the precibarium of *H. vitripennis*, thus, distal to the closed valve and inside an invagination at the base of the valve. These findings support the crucial role of vector anatomy in the bacterial inoculation process, via the two different types of egestion that we modeled.

## Materials and methods

### Plant and insect rearing

 *G. atropunctata* were reared on basil, *Ocimum basilicum* (L.) (Johnny’s Selected Seeds, Waterville, Maine) as fodder plants. Basil was grown from seed in a USDA greenhouse under natural light supplemented with artificial lights for a 14:10 (L: D) photoperiod with temperatures from 24 to 29 °C. Insects were kept on fodder basil in colony cages inside an indoor rearing room in a USDA insectary under artificial lights at 16:8 (L: D) photoperiod and ~ 25 °C, as described in further detail in^21^. Plants in colonies were changed weekly to ensure that insect functional foreguts were *Xf*-free prior to dissection. Field-collected *H. vitripennis* were swept from cultivated ornamental plants (chiefly *Photinia* spp.) along roadways in Bakersfield, CA, in late summer and transported directly to a greenhouse in the USDA ARS containment facility in Parlier. Insects were maintained with supplemental lighting at 16:8 (L: D) photoperiod at ~ 27 °C on a mixture of cowpea, *Vigna unguiculata* (L.), basil, and sunflower, *Helianthus annuus* (L.), plants (also from Johnny’s Selected Seeds, Waterville, Maine) in the same cage^22^until prepared for microscopy.

### Light microscopy

 Three insects for each of the two study species were rapidly anesthetized to near-death with CO_2_ before the heads were excised and placed in centrifuge tubes holding tissue-stabilizing, ethanol-based hand sanitizer. Samples were then overnight-shipped to the Laudier laboratory for further processing. Hand sanitizer was used to stabilize tissue and to prevent necrosis during transport, as well as for a long-term storage solution at 4^o^ C. Samples were removed from the sanitizer and placed into tissue processing cassettes. Tissue samples were rinsed well with water and placed into Acetic acid Zinc Formalin fixative (AZF) for 72 h. Post-fixation, samples were dehydrated with a combination of 2-ethoxyethanol and acetonitrile. Post-dehydration, samples were cleared with benzyl benzoate^23,24^then processed to and embedded in a proprietary hydrophobic acrylic resin similar to Technovit or other hydrophobic resin recipe. Two-micron-thick sections were cut using a Leica rotary microtome (RM 2165, Wetzlar, Germany) from embedded blocks and individually mounted on numbered adhesive microscope slides, allowing for sequential identification. Selected sections were deplasticized then stained with hematoxylin and counter-stained with eosin^25^. Hematoxylin (blue stain) has a strong affinity for nucleic acids, which has been used for decades to identify microbes and cell nuclei^23^. Eosin (pink stain) has a strong affinity for proteins and is used to identify resilin-containing cuticle and cellular components in insects^23^. Slides were examined and imaged using a Zeiss AxioImager Z1 compound light microscope (Oberkochen, Germany). Images were enhanced for contrast, when needed, using Adobe Photo Shop 2024. Images presented are from one, representative individual from each species.

### Scanning electron microscopy

 Previously unpublished views of epipharyngeal precibaria from 40 *G. atropunctata* from a recent study^16^ were examined for the present study. The five insects chosen for figures showed the most representative views of different stages of opening or closing of the precibarial valve. Detailed SEM preparation methods were previously published^16^. In brief, *G. atropunctata* were put in a 4^o^ C freezer for 1–2 min prior to excision of heads under 4% glutaraldehyde and fixation for 24 h under vacuum. Samples were subjected to a standard ethanol dehydration series then critical-point dried with ethanol as the transitional solvent (Tousimis Autosamdri-815B critical point dryer, Tousimis Co., Rockville, MD, U.S.A.). Heads were dissected to expose the opened halves of the precibarium, sputter-coated with palladium-gold using a 30,300 Model Lads sputter coater (Williston, VT, U.S.A.), then viewed under a Hitachi S4000 scanning electron microscope (Hitachi-High Technologies America, Schaumburg, IL, U.S.A.)

## Results

A detailed, 3D model of the precibarial structures in *G. atropunctata* was previously published, in which the terminology of most parts therein was standardized^13^. The present paper primarily uses those terms, when applicable. However, because findings herein are compared with those from an earlier study of *P. spumarius*^12^terms from that paper are synonymized in italics. In addition, Table 1 describes four cuticular structures comprising the precibarial valve and surrounds that have not been previously named. Because they are critical to understanding valve operation, these terms and descriptions should be understood before reading on. Also, all descriptions of micrographs herein are discussed in relation to orientation in the figure referenced; direction (distal versus proximal) will not always be stated.

**Table 1:** Definitions of new terms to describe the precibarial valve assembly and surrounds, and key to the segments in Fig. 2C.

Segment number in Fig. 2C	Description	Terms used herein	Ruschioni terms
1	Cuticle at the top of the basin. Left (proximal) 2/3 is elastic with resilin; right (distal) 1/3 is stiffened with sclerotin.	Basin	*Basin-like structure* (*Bls*)
2	Bifurcated tips of the tendon of the precibarial valve. The thin tips are elastic where they attach to the stiffened parts of the basin cuticle (left branch) or the middle of the valve (right branch).	Tendon tips	none
3	A flexible strip of cuticle that is thin in section and connects the inner, elastic end of the valve to the elastic edge of the cuticular pad. This edge is part of both the joiner and pad cuticles. The invagination at the bottom of the precibarial pit/ring (*Bsi*) is attached to the joiner cuticle.	Joiner cuticle *Bell-shaped invagination* (*Bsi*)	none *Bell-shaped invagination* (*Bsi*)
4	An enlarged, globular (in 3D) pad of procuticle between the valve and the distal enclosure. The part closest to the lumen of precibarium is part of the joiner cuticle. The middle of the pad is strongly stiffened with sclerotin, while the part furthest from the lumen is elastic with resilin.	Cuticular pad	*Procuticle* (*Prc*)
2, 3, 4 and valve	The functional unit of the precibarial valve, composed of the tendon tips, valve (with its flap), joiner cuticle, and cuticular pad. The operational mechanism hypothesized herein uses all of these parts.	Valve assembly	none

### **Blue-green sharpshooter**, ***G. atropunctata***: **light micrographs**

**Fig. 2:**
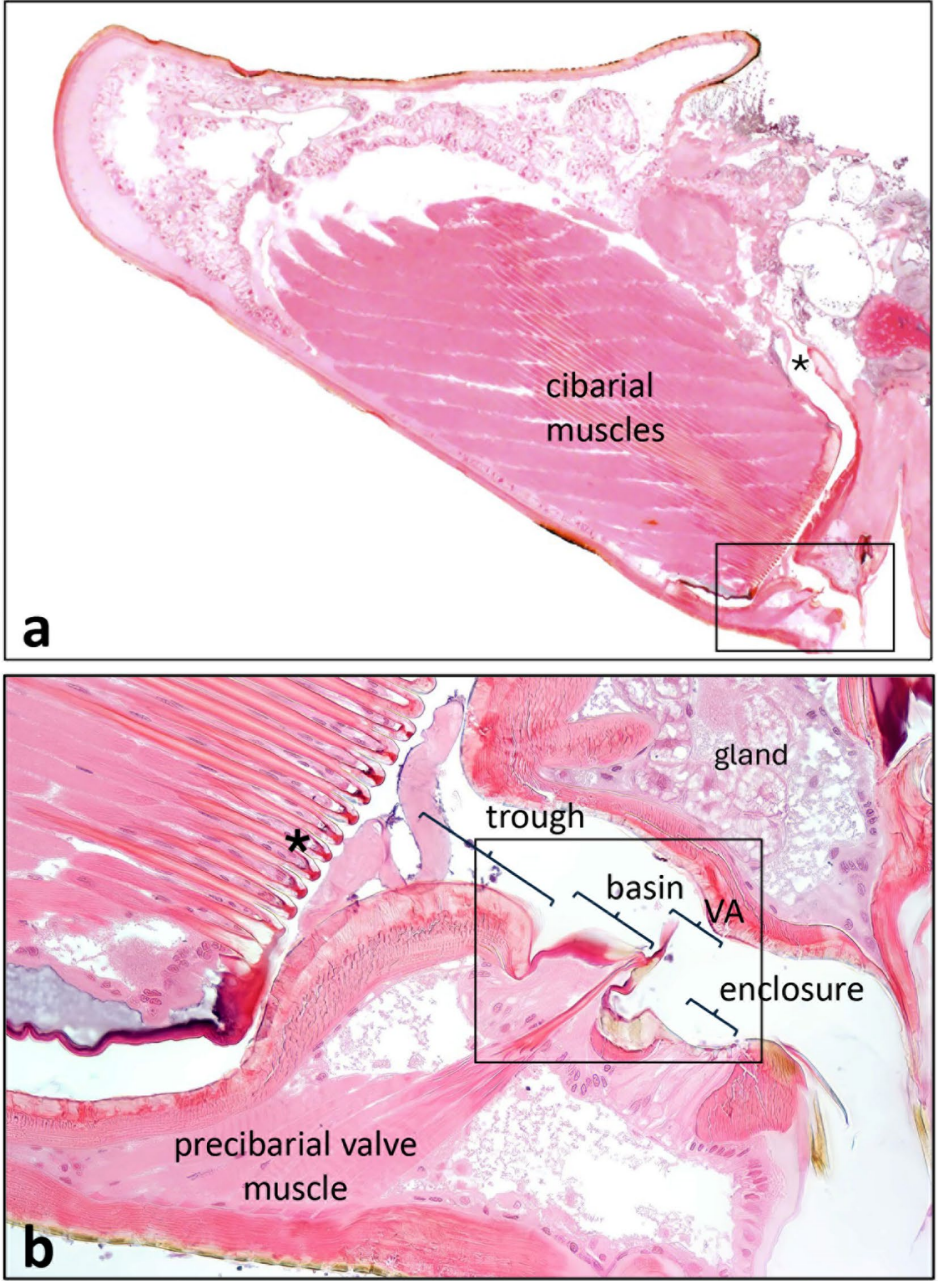
Overview of the sharpshooter cibarium and precibarium. (**a**) Saggital section through the head of *G. atropunctata* showing the location of the small precibarium (boxed area, lower right, enlarged in part **b** ) in relation to the large cibarial muscles and shape/size of the head. (**b**) Enlarged view of box from part **a** , with the four regions (trough, basin, valve assembly [VA], distal enclosure [abbreviated enclosure]) of the epipharyngeal half of the precibarium. The full length of the precibarial valve muscle is also shown, as well as the unknown gland previously described in *P. spumarius*^12^. Boldly boxed area in part **b** is expanded in Fig. 3. *, opening to the true mouth. Note that structures in Fig. 2 and subsequent figures are mirror images of those in Fig. 1. For color version: colors are based on those in Fig. 2, that is, red, resilin; yellow, sclerotin; pink; procuticle. For black-and-white version: dark gray, resilin; pale gray, sclerotin; medium gray, procuticle.

 A large part of sharpshooter heads is filled with the cibarial muscles (Figs. 1 and 2a), which insert on the lid-like diaphragm of the cibarium in the form of multiple arches (Fig. 2b, asterisk). The cibarium is tilted about 45 degrees from vertical in Fig. 2a and, like all remaining figures, is a mirror image of Fig. 1. Fluids, when swallowed (ingested) flow through the true mouth^33^ (Fig. 2a, asterisk). Distal to the cibarium (towards the lower right in Fig. 2a) lies the very small precibarium, a short canal that conveys fluid from the food canal in the stylets to the cibarium. The four parts of the epipharyngeal side of the precibarium (trough, basin, valve assembly [VA], and distal enclosure [enclosure for short]) are labelled in Fig. 2b; the term “valve assembly” is new to this paper. The basin (*basin-like structure* or *Bls*) houses the proximal (P-) chemosensilla (*proximal group or PG*), while the distal enclosure houses the distal (or D-) chemosensilla (*distal group or DG*); the precibarial valve separates these groups of sensilla. The muscle that operates the valve is clearly separate from the cibarial muscles (Fig. 2b).

**Fig. 3:**
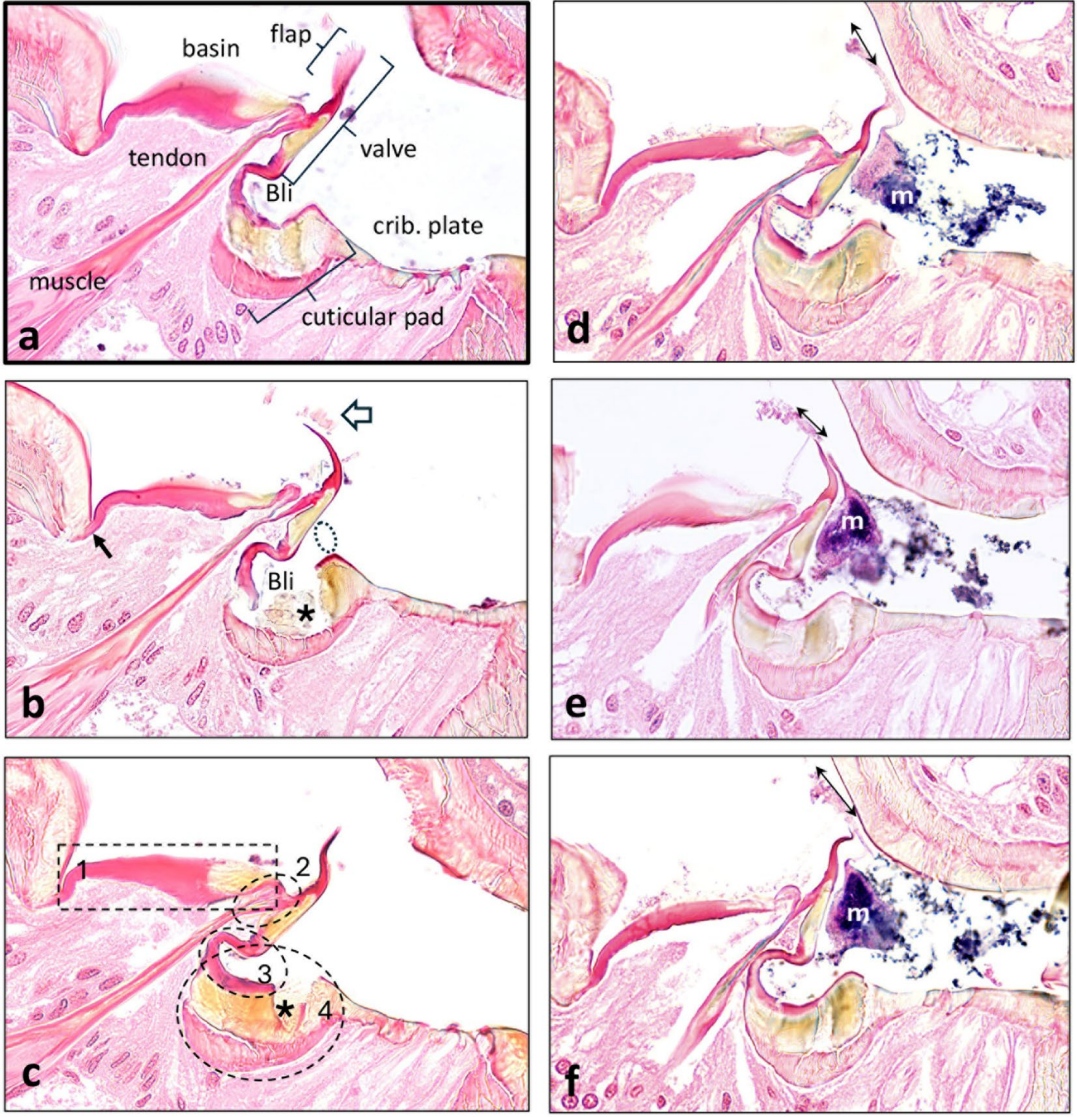
Close-ups of sections through the sharpshooter precibarial valve. (**a** – **c**), *G. atropunctata*, all contiguous sections comprising the valve; (**d** – **f**), *H. vitripennis*, three out of six sections comprising the valve. (**a**) First section housing the valve assembly of *G. atropunctata*, labelled to show important parts. This section is an enlargement of the black-boxed area in Fig. 2b , hence bolder outline. At the top, the densely-staining cuticular tendon inserts into the attachments to the valve and interlaces at the bottom with the fibers of the muscle. Basin (*Bls*, *basin-like structure)*; *, location of the pit/ring (*Ring*) at the mouth of the *bell-like invagination* (*Bli*); crib. plate, cribriform plate of the distal (D-) sensilla in the distal enclosure. (**b**) Second section housing the valve assembly. This is a particularly clear view of the precibarial valve muscle’s tendon bifurcating at the distal end into two tendon tips attached to the right side of the basin cuticle (left tip) and middle of the valve (right tip). The left tendon tip is very narrow and folded over at its attachment to the basin cuticle. (**c**) Third section housing the valve assembly. Dotted, numbered boxes correspond to the numbered segments described in the narrative and Table 1. (d) Structures of the epipharyngeal precibarium in *H. vitripennis* are nearly identical to those seen in *G. atropunctata* (**a** – **c**). The primary difference is the triangular appearance of the tendon attachment to the basin cuticle and valve. However, this is likely due to the closely appressed position of the tendon tips because the same area takes on a more bifurcated structure in subsequent sections (**e**) and (**f**), similar to that of *G. atropunctata*. The most striking difference in these images compared with those in a – c is the presence of unknown particles (presumed microbial biofilm) (white m) distal to the valve (to the right of it, in the image) in the distal enclosure, seen in all three sections. See text for more information on putative microbes. For color version: colors are based on those in Fig. 2, that is, red, resilin; yellow, sclerotin; pink; procuticle. For black-and-white version: dark gray, resilin; pale gray, sclerotin; medium gray, procuticle.

Figure 3 shows closer views of three thin sections that comprise the precibarium in *G. atropunctata* (left side images, Figs. 3a, b, c) and three more from *H. vitripennis* (right sight images, Figs. 3d, e, f). It is structurally relevant that cuticle in the basin and valve assembly stained differently than that in the rest of the precibarium, which stained pale pinkish in color images (Figs. 2a, b). This occurred because the cuticular proteins resilin and sclerotin, both developed during post-molting tanning, differentially absorb eosin. Dark red coloring indicates resilin wherein the proteins are cross-linked with tyrosine, making them elastic. Conversely, sclerotin results when proteins are cross-linked and hardened/melanized with phenolics such as catecholamines, making them stiff. Sclerotin does not absorb eosin well but retains its melanized yellow-orange color^23^. That said, some blue-ish color overlaid parts of the right-side images because blue was enhanced for those images. Thus, we infer that dark red coloration represents a highly elastic, resilin-containing structure, whereas yellow-orange represents a stiffer, sclerotin-containing structure.

The precibarial valve muscle terminates in a narrow, distinct and solid tendon, which has a high resilin content on its edges and a sclerotin center (Fig. 3a, b, c). As labelled in Fig. 3a, the full length of the valve is a long structure with 2/3 quite solid; the upper 1/3 (the *flap* [*Flp*]) appears to be flexible because it is in a different position or aspect in each thin section and contains a high amount of resilin (Figs. 3a, b, c). The tendon attaches to the basin and valve assembly, which are complex structures explained in Table 1; Fig. 3a (labelled with names) and Fig. 3c (labelled with numbered segments, explained below). The basin is on the left side and is either thin and straight (Fig. 3b) or thick and bulging upward (Figs. 3a and c). These bulges are more visible in SEM views, described below. The valve assembly is composed of the valve (with flap), tendon tips, joiner cuticle, and cuticular pad (Fig. 3a**)**.

Remarkably, and very differently than interpreted for *P. spumarius*^12^there are three cuticular attachments to the precibarial valve. The first two are from the bifurcated (forked) ends of the tendon (termed tendon tips), best seen in Fig. 3b, and numbered segment 2 in Fig. 3c; Table 1. The left tendon tip is attached to a thin extension of the basin cuticle (the latter being segment 1 in Fig. 3c; Table 1) so that the basin extension and the tendon tip are folded together. The right tendon tip is attached to the middle of the valve. Thus, the valve is indirectly attached to the basin cuticle on its upper (in the image) end and directly attached to the tendon (thence muscle) in the middle. The third attachment of the valve is at its distal (lower in the image) end, to the joiner cuticle (segment 3 in Fig. 3c; Table 1) on the lumen side of the cuticular pad or *Procuticle* (*Prc*) (segment 4 in Fig. 3c; Table 1). As a result of these three attachments in four segments, the precibarial valve is double-hinged: (1) at the middle of the valve to the basin cuticle and valve muscle via the two tendon tips, and (2) at the inner end of the valve to the joiner cuticle that is braced by the cuticular pad.

Interestingly, the middle of the valve (where it attaches to the right tendon tip) is sclerotized, thus relatively stiff. Likewise, the right end of the basin cuticle that attaches to the left tendon tip is also sclerotized and stiff. In between, the tendon tips are flexible with resilin. Thus, the elastic tendon tips can flex around the stiffer basin cuticle and middle of the valve; they can also move apart/separate (splay) or move together/adjoin. Mechanically speaking, this anatomy supports that the sclerotized cuticle in the middle of the valve (just below the attachment of the right tendon tip) can act like a fulcrum, around which the valve can pivot. The left 2/3 of the basin cuticle is also flexible, with red-staining resilin. Thus, that section of cuticle can flex and bulge.

Directly below the valve and surrounded by the joiner cuticle and cuticular pad is the *bell-like invagination* (*Bli*) (Fig. 3a). Only a small portion of this invagination is open to the lumen of the precibarium (through the precibarial pit/ring [*Ring*], indicated with a dotted ring in Fig. 3b). Mechanically, it is likely that the sclerotized section of the cuticular pad acts like an anchor to hold the valve above it in place, with flexible attachments that flex into (in the case of the joiner cuticle) or cushion (in the case of the inner/lower layer of the cuticular pad) the valve movements.

The appearance of the basin and valve assembly described above indicates that the muscle is relaxed in this view, not contracted, because (1) the tendon tips are close together and relaxed, and (2) the tendon is not stiff and straight; it is pushed in slightly to the left by the bend of the joiner cuticle. Accordingly, the relaxed state of the basin-valve assembly is *closure*, in agreement with the conclusion for *P. spumarius*^12^. However, the finding that the valve is hinged in the middle and therefore the end must flip “upward” to close is distinctly different from the operational mechanism proposed for *P. spumarius*^12^ (see Discussion).

### **Blue-green sharpshooter**, ***G. atropunctata***: **scanning electron micrographs**

**Fig. 4:**
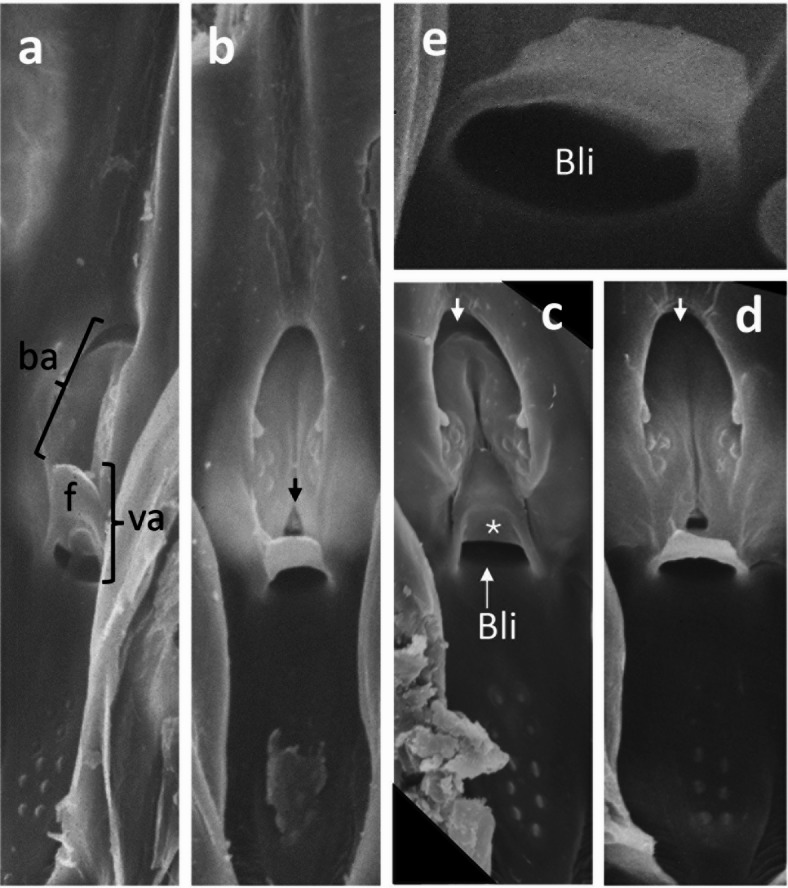
Various degrees of closure for precibarial valves viewed from the lumen interior of the precibarim. Previously unpublished, SEM images of five *G. atropunctata precibaria* from the study of^16^. (**a**) *Mostly opened valve* in an oblique view of the epipharyngeal half of precibarium. This view reveals the rounded, bulbous aspect of the basin (ba) and valve assembly (va) including valve flap (f). (**b**) *Valve mostly closed* in a straight-on view of the epipharynx; the small area between the arms of the bifurcated tendon attachment is visible through a hole (black arrowhead). (**c**) *Fully opened valve* showing how the valve fits snugly into its triangular receptable in the basin. The precibarial pit/ring (*Ring*) opening of the *bell-shaped invagination* (*Bli*) has been bent into a smaller opening pointed upward (in the image). (**d**) *Fully closed valve* with the pit/ring (*Ring*) tilted strongly forward toward the distal enclosure. White arrows denote the proximal groove around the basin; note that the groove is sharply delineated in part c but spread out in part d. (**e**) Close-up view from a fifth insect showing the distinctly ring-like structure of the opening to the bell-like invagination (*Bli*).

 To further clarify the operational mechanism of the valve, it is useful to examine the view from the interior lumen of the precibarium when the epipharynx and hypopharynx have been separated by dissection and individually imaged via SEM. Figure 4 shows epipharyngeal precibaria from five out of 40 *G. atropunctata* imaged^16^. The chilling process prior to dissection described in Methods (similar to that used for *P. spumarius*^12^ resulted in varying degrees of closure of the precibarial valve, with valves partially open (Fig. 4a), partially closed (Fig. 4b), fully open (Fig. 4c), and fully closed (Fig. 4d). All views show the characteristic double-bulge shape of the epipharyngeal basin (*Bls*) (labelled in Fig. 4a) with a longitudinal, medial groove between the bulges. Each bulge of the basin cuticle in SEM views corresponds to each sectioned view through a bulge of the basin in Figs. 3a and c. The medial groove or sides of the basin correspond to thinner cuticle (Fig. 3b). Thus, the right sides of the basin and/or the medial groove are not very deeply grooved relative to the lumen. This is quite different from the left-side edge of the basin in Fig. 3b (black arrow), which comprises a deep trench in the proximal (“top edge”) of the basin cuticle in the lumen view seen from “above” in Figs. 4c and d (white arrows). It is important to note that, while the proximal groove around the basin is always nearly black (thus, deep) in the SEM view, the basin bulges themselves are more rounded/when the valve is fully open (Fig. 4c) but flattened and deeply shadowed when the valve is fully closed (Fig. 4d).

SEM views reveal a more three-dimensional aspect than sectioned views. This is especially notable in Fig. 4a, which shows an oblique angle of view of the epipharyngeal plate that makes up the precibarium. Figure 4a demonstrates the overall rounded appearance of the basin and valve assembly, which appears to protrude out of the otherwise flattish/gently curving length of the precibarium when the valve is mostly open (also seen in Fig. 4c with the valve fully opened). This relative flatness is suggested by the lack of contrast between the trough (upper part of Fig. 4a) and the distal enclosure (lower part of Fig. 4a). Differently, Figs. 4b and d show stronger contrast, with the distal enclosure cloaked in darkness. When the valve is closed, the precibarium demonstrates a step-like side view (similar to the sectioned view in Fig. 2) wherein the basin-valve assembly is embedded within the wall of the step downward (near the dotted ring in Fig. 2b). Thus, the strong depth of SEM image conveys the 3-dimensional aspect also seen in longitudinal LM sections.

Comparing LM and SEM images reveals that when the valve is closed only the flap of the valve is visible in SEM aspect (Fig. 4d). The remainder of the valve becomes tilted inward and hidden within the depth of the precibarial pit/ring (*Ring*), which becomes the prominent part of the “downward” 3D step. When closed, the opening to the *bell-like invagination* (*Bli*) takes on the appearance of a ring-like collar (*Ring*) (Fig. 4e). It is important to note that the basin (*Bls*) does not become inverted and convex when the valve is open (as suggested for *P. spumarius*^12^, although the appearance of the basin is flatter (Fig. 4c). The basin bulges housing the P-sensilla and the central cleft are intact. These changes in aspect of the basin are important for understanding the operational mechanism of the precibarial valve (see Discussion).

Separate from the above points, the SEM views reveal the following additional properties relevant to a hypothesized valve opening mechanism. (1) The flap of the valve is triangular in shape, leaving space for fluid to flow around its side in a circular tube. It also nestles into a triangular depression at the end of the medial grove in the basin, exposing part of the stiffened portion of the valve, but not all of it (Fig. 4c, white asterisk). (2) The length of the valve is longest in the middle, leaving space along the sides when the flap closes into a rounded, arch-like area “above” the valve formed by the hypopharyngeal surface. (3) The pit/ring opening of the *Bli* changes configuration when the edge of the flap flattens and is drawn out to fit into the triangular depression. This action changes the opening into more of an archway than a ring (compare Figs. 4c, d, e). (4) Grooves along the walls of the basin become deeper and the bulges therein taller and accentuated when the valve is open rather than closed (compare Figs. 4c and d, respectively). (5) Finally, a small hole is visible beneath the valve when it is closed, with a small cuticular structure viewed therein (see in Figs. 4b [black arrow] and d). We surmise that the cuticular structure in the hole is part of one of the tendon tips; the hole allows separation or adjoining of the tendon tips.

### **Glassy-winged sharpshooter**, ***H. vitripennis***: **light micrographs**

 The right side of Fig. 3 (d,e, and f) shows three out of the six thin sections that comprise the precibarial valve in *H. vitripennis*, a larger insect than *G. atropunctata*. The views show almost all the same regions and appearance of the precibarium (that is, trough, basin, valve assembly, and distal enclosure) as for *G. atropunctata*. The only exception is that two sections (Fig. 3d, f) show the basin cuticle very thin and extended, therefore comprising the longitudinal medial groove or basin side edges. In contrast, Fig. 3e shows the basin cuticle bulging, comprising one bulge of the basin. Note also that the precibarial valve muscle is not entirely visible in Figs. 3d and e, although the tendon is intact. We hypothesize that the muscle has been moved out of the plane of view of that section, also supporting that the muscle is relaxed and flaccid when closed. Thus, two unrelated species of sharpshooters have identical morphology of the precibarial basin and valve assembly.

The most striking feature of Fig. 3 right-side images is the presence of blue-stained flocculent patches inside the lumen of the precibarium, to the right side of the image. The valve is closed and these flocculent patches (likely biofilm of unidentified microbes due to affinity for nuclei acids) are suspended within the area distal to the valve, including a few inside the *bell-shaped invagination* (*Bsi*). Even more remarkable is the distinct presence of a flimsy trail of material directly flowing from the microbial patch to the other side of the slightly open flap of the precibarial valve. Because tissues were alcohol-stabilized prior to shipping, this flimsy trail would not have developed or moved during shipping of the samples. This trail (as well as anatomical features described above) strongly supports that the flap portion of the valve does not fit tightly against the opposite cuticular wall, and that the valve is “leaky,” especially on its sides (Fig. 3d, f).

By comparing the position of this trail and the flap in each image (Fig. 3d, e, f), in relation to the opposite area of hypopharyngeal cuticle, one can also discern a challenge with sectioning through such densely cuticular, tiny structures. The edge of the resin section (denoted by a double-headed arrow in Figs. 3d, e, f) containing the microbes is separated slightly from the opposite cuticle, revealing different distances between the flap and opposite cuticle for each section. Close examination of the *G. atropunctata* sections in Fig. 3a, b, and c shows similar disparities. Each of the six sections pictured in Fig. 3 is slightly different, thus indicating separation during sectioning, a common artifact of such sectioning. Nonetheless, the microbial trail and the apparent edge of the separated thin section show that the hypopharyngeal cuticle was positioned very close to the tip of the valve, as in Fig. 3f. In addition, small chunks of cuticle remain adhered to the flap section in Fig. 3b (hollow arrow). Thus, the valve closed against a rounded edge of hypopharyngeal cuticle (as also shown in^12^see more on this below).

Three characteristics demonstrate that the flap curves and waves in the strong currents of fluid flow through the precibarium: (1) the fortuitous presence of the roughly triangular shape of the microbial mass leading into (2) the trail of matter around the valve, combined with (3) the different curvature of the flap in each section. Current flow was so strong (as previously demonstrated by hydrodynamic measurements^13)^ that it shaped the microbial biofilm and microbial trail so that it appears drawn inward. Interestingly, the cibarial muscles of the *H. vitripennis* head portrayed in the right-side images in Fig. 3d, e, and f were elevated asymmetrically, indicating that the insect probably was swallowing (dropping the diaphragm) when it died (see Supplementary **Fig. S2b** and discussion). However, the diaphragm would have been repeatedly withdrawn prior to each swallow, which may have “frozen” the appearance of the microbial trail. In contrast, muscles of the *G. atropunctata* head portrayed in the left-side images in Fig. 3a, b, and c had completely fallen into the cibarium (see in Supplementary **Fig. S2a**). In addition, the strong curve of the flap to the left in Fig. 3b and e, compared with the smaller, S-shaped bend of the flap in Figs. 3c, d, and f, indicate that the valve leaks more strongly towards the cibarium (to the left) rather than outward (to the right). Finally, Figs. 3c and d indicate that the flap is triangular in shape with its longest length at the tip of the triangle shown in Fig. 3b. The long flap tip lays closely against the hypopharyngeal cuticle in Fig. 3b.To either side, the flap is shorter in Figs. 3c and d, and in Figs. 3a, c, d, e, and f. Yet, the tips of the flap barely touched the cuticle, implying that fluid could pass more easily to either side of the flap. This triangular shape is the same as what is seen in the SEM view in Fig. 4c.

When the cibarial dilator muscles withdraw the diaphragm, fluid is sucked inward from the stylets through the precibarium. When closure of the valve occurs, its flap tip tightly adjoins the hypopharynx but the flap sides are open. This allows a small amount of fluid to leak through the sides of the flap into the proximal part of the precibarium and cibarium. Accordingly, anatomical evidence supports that the precibarial valve is both hinged and leaky in operation.

## Discussion

### Comparison of precibarial anatomy for spittlebug and sharpshooters

**Fig. 5:**
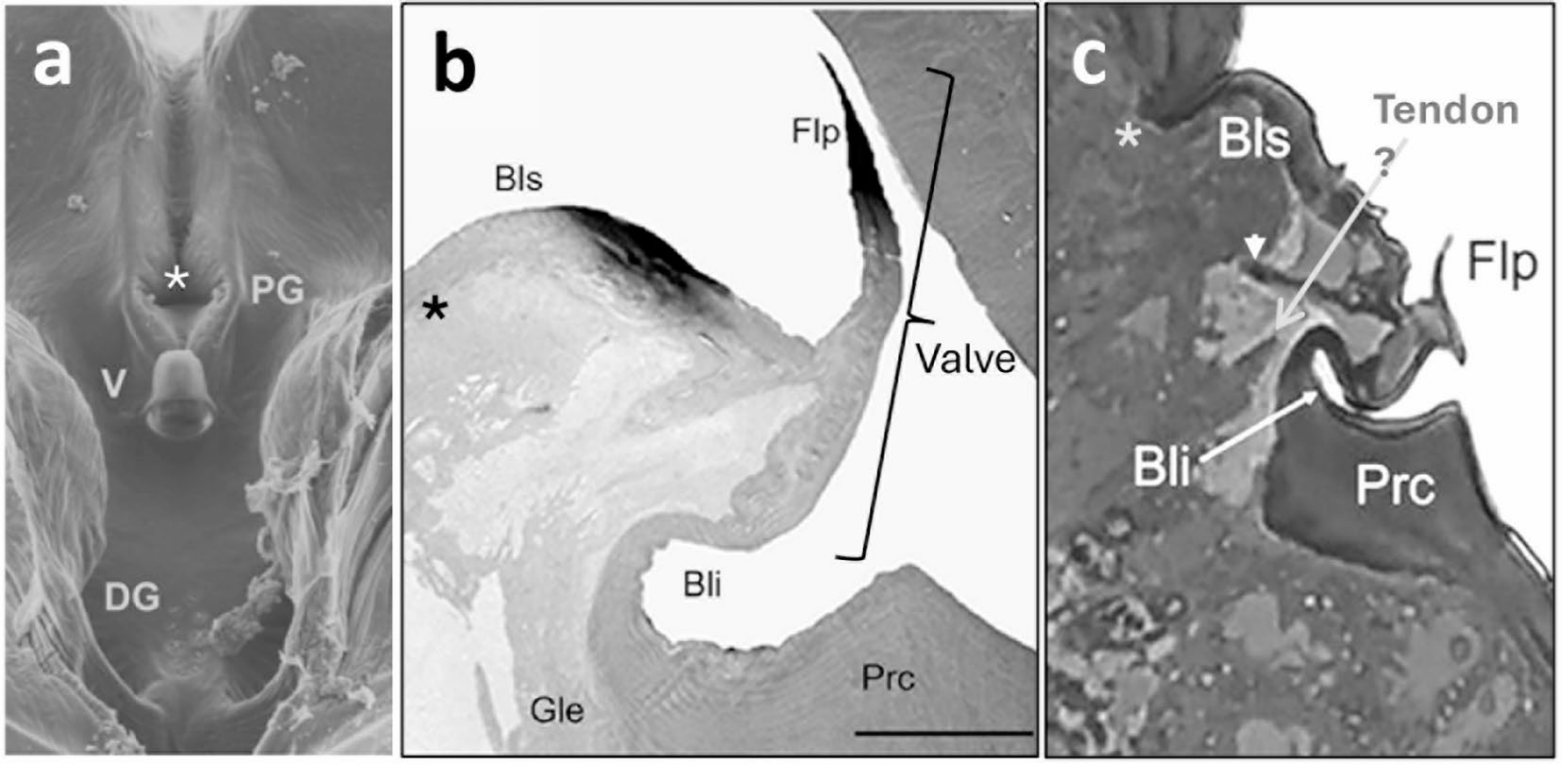
**Previously published views of the precibarium and its valve in** ***Philaenus spumarius***. (**a**) Scanning electron micrograph of the epipharyngeal half of the precibarium viewed from the lumen, showing the same four parts (trough, basin, valve assembly, and distal enclosure [top to bottom]) as in sharpshooters. From^19^Fig. 5a. White asterisk was added for the present paper. (**b**) Transmission electron micrograph through the precibarial valve while closed. From^12^Fig. 1C. Black asterisk, bracket and “Valve” labels were added for the present paper. Scale bar, 25 μm. (**c**) Transmission electron micrograph through the precibarial valve while putatively open. From^12^Fig. 1B. Bli, *bell-like invagination*; Bls, *basin-like section*; Flp, *flap*; DG, *distal group of sensilla*; Gle, glandular epithelium; PG, *proximal group of sensilla*; Prc, *procuticle*. Gray asterisk, horizontal medial groove from lumen; V, valve. Gray asterisk, “Valve,” white arrowhead and “Tendon?” labels were added for present paper. Reproduced with permission of the publisher via Copyright Central.

 A scanning electron micrograph of the lumen structures in *P. spumarius*^19^ (Fig. 5a) shows great similarity to those in sharpshooters. The precibarium is divided into the same four regions (trough, basin, valve assembly, and distal enclosure; from top to bottom of Fig. 5a, originally from^12^. The precibarial valve is flat and flap-like as in other auchenorrhynchans (hoppers)^15,20^ rather than piston-like as in sternorrhynchans (aphids, whiteflies, and their relatives)^26–29,32^. The only discernible differences between sharpshooters and *P. spumarius* in SEM views are that: (1) the medial cleft in the basin is horizontal, or perpendicular to the long axis of the precibarium (Fig. 5, white asterisk added for this paper) in *P. spumarius*, rather than vertical (parallel to the long axis) as in sharpshooters, and (2) the proximal edge of the basin is not indented in *P. spumarius*, as in sharpshooters.

Transmission electron micrographs (TEM images) of the interior of the basin and valve assembly in *P. spumarius*^12^ (Figs. 5b, c) show some similarities with LM sharpshooter images herein of the same structure. Figures 5b and c show two out of three images in^12^; the third TEM image in^12^ is nearly identical to Fig. 5b herein, therefore it was not re-published here. Similarities between sharpshooters and *P. spumarius* include: (1) the convex, bulging silhouette of the basin (*Bls*) is protruding into the precibarium lumen (Fig. 5b) and (2) the *bell-like invagination* (*Bli*) is present and attached to the joiner and the cuticular pad of *procuticle* (*Prc*) (Fig. 5b, c). Differently from sharpshooters, there does not appear to be a deep edge between the basin and trough in Fig. 5a; this suggests that the deep depression in Figs. 5b and c (black and gray asterisks added, respectively) must represent the horizontal median cleft in the basin. Thus, the cleft is relatively shallow and not attached to the precibarial valve tendon. The most important structure, the precibarial valve itself, however, is challenging to interpret.

The cuticle of the closed precibarial valve in *P. spumarius*^12^ (bracket and “valve” label added to Fig. 5b) appears very similar to the area labelled ‘valve’ in Fig. 3a herein. However, the valve/flap (*Flp*) structure in Fig. 5c bears no resemblance to any image in the present study, nor to the other two images of *P. spumarius* in the same paper^12^. The pi-shaped flap (*Flp*) in Fig. 5c appears to be open, but it seems too small (in relation to the basin) compared to the flap (*Flp*) in Fig. 5b. Also, the hook-shaped protuberance end of the flap in the right/lower (in the image) of Fig. 5b is unlike anything seen in other images in either study. Careful examination of the image discerns a rough black line (Fig. 5c, white arrowhead added); this line does not appear to represent any known structure. Because it aligns with the right-side protuberance, we suspect that this line represents a fold in the plastic section, resulting in crumpling and foreshortening of the valve assembly, causing the flap to appear open when it is likely to have been at least partially closed. This is the most logical explanation for the very short length of the valve/flap in Fig. 5c relative to the same structure in Fig. 5b. In addition, the joiner cuticle surrounding the *Bli* is squeezed downward, a position we never saw in the present images.

Most crucially, the location and attachment(s) of the valve muscle and its tendon are not clearly shown in any published images of *P. spumarius*^12^. It is claimed that the valve muscle and its attachment (*basin-like muscle*, *Blm*) were imaged as the very fuzzy structure (Supplementary **Fig. S3**). However, this “muscle” is actually attached to the edge of the cuticular lumen not near an invagination and it is likely to be cuticular (not muscle), perhaps part of the joiner cuticle. The actual valve attachment is not visible. Yet, it is indispensable for understanding the mechanism of valve operation to visualize a clear view of the attachment of the muscle that moves the valve. It is especially important to determine whether the attachment forms a hinge directly on the valve itself or on an adjacent invagination. It may be possible that a triangular extension of tissue in Fig. 5c (added gray arrow and label “Tendon?”) was a tangential slice of the muscle and its tendon. However, this tissue does not look cuticular, as does the sharpshooter tendon in images herein.

Despite lack of any unambiguous micrograph showing attachment of the valve muscle/tendon to any structure in *P. spumarius*, the published model for valve operation in that species^12^ (Supplementary **Fig. S4A**) suggests otherwise. It indicates that the muscle attaches directly to the wall of a large (but invisible and unlabeled in micrographs) invagination proximal to the basin (*Bls*) lying directly under the flap of a closed valve. However, the microscopic section through a closed valve (Fig. 5b) does not show this invagination^12^. The diagram also indicates that this invagination (“Bls”) enlarges during valve opening as the basin (*Bls*) completely inverts, becoming concave instead of convex with respect to the precibarial lumen. Yet, the only sectioned image through an “open” valve (Fig. 5c) shows no such inverted convex basin/*Bls*, resulting invagination, nor muscle/tendon attachment^12^. Neither can the source of this invagination be the horizontal median cleft of the basin, and the somewhat convex shape of the basin itself. Internally, the cleft is shallow, does not form an invagination, and does not have a tendon/muscle attached to it (Figs. 5b, c).

Furthermore, the valve closing mechanism of *P. spumarius* in the diagram in Supplementary **Fig. S4B** shows that the valve does not hinge but instead is indirectly pushed upward to flatly appress against the hypopharyngeal cuticle^12^. There is evidence that the unhinged, flat surface of the tip of the valve flap closes flat against the hypopharyngeal cuticle of both sharpshooters (Fig. 3b**)** and *P. spumarius* **(**Fig. 5b**)**, but such a similarity is not shown. Instead, Supplementary **Fig. S4b** diagrams the *P. spumarius* pit/ring (*Ring*) with the edge touching the hypopharyngeal cuticle, yet no micrograph provides evidence for this. Instead, the opening of the pit/ring is smaller in sharpshooter images herein than diagrammed in the spittlebug model^12^. The edge of the pit/ring in sharpshooters is also far from the hypopharynx (indicated in Fig. 3b by a dotted ring). While the sharpshooter pit/ring does rotate to face forward/distally when the valve is closed, it does not completely fill the lumen of the precibarium; the valve blocks the lumen, not the pit/ring. The means by which the valve is closed in the *P. spumarius* model is also counter-intuitive because it would completely block inward suction from the cibarium as it withdraws, thereby preventing fluid flow into the *bell-like invagination* (*Bli*) that is proposed in **Fig. S4C**. Accordingly, without an invagination, convex basin/*Bsl*, and valve connection, the published evidence for the valve operational mechanism of *P. spumarius*^12^ provides only inconclusive support for the authors’ model.

We conclude from this detailed comparison that evidence for the published model for operation of the *P. spumarius* precibarial valve^12^ is inconclusive, does not apply to sharpshooters, and may not be accurate for spittlebugs. The crucial missing evidence is a clear means of attachment of the valve muscle/tendon to the valve, whether it is direct and hinged (as definitively shown for sharpshooters herein) or indirect and not hinged (as suggested, but not demonstrated, in the latter authors’ model).

### Hypothesized model for operational mechanism: Closing the valve

**Fig. 6:**
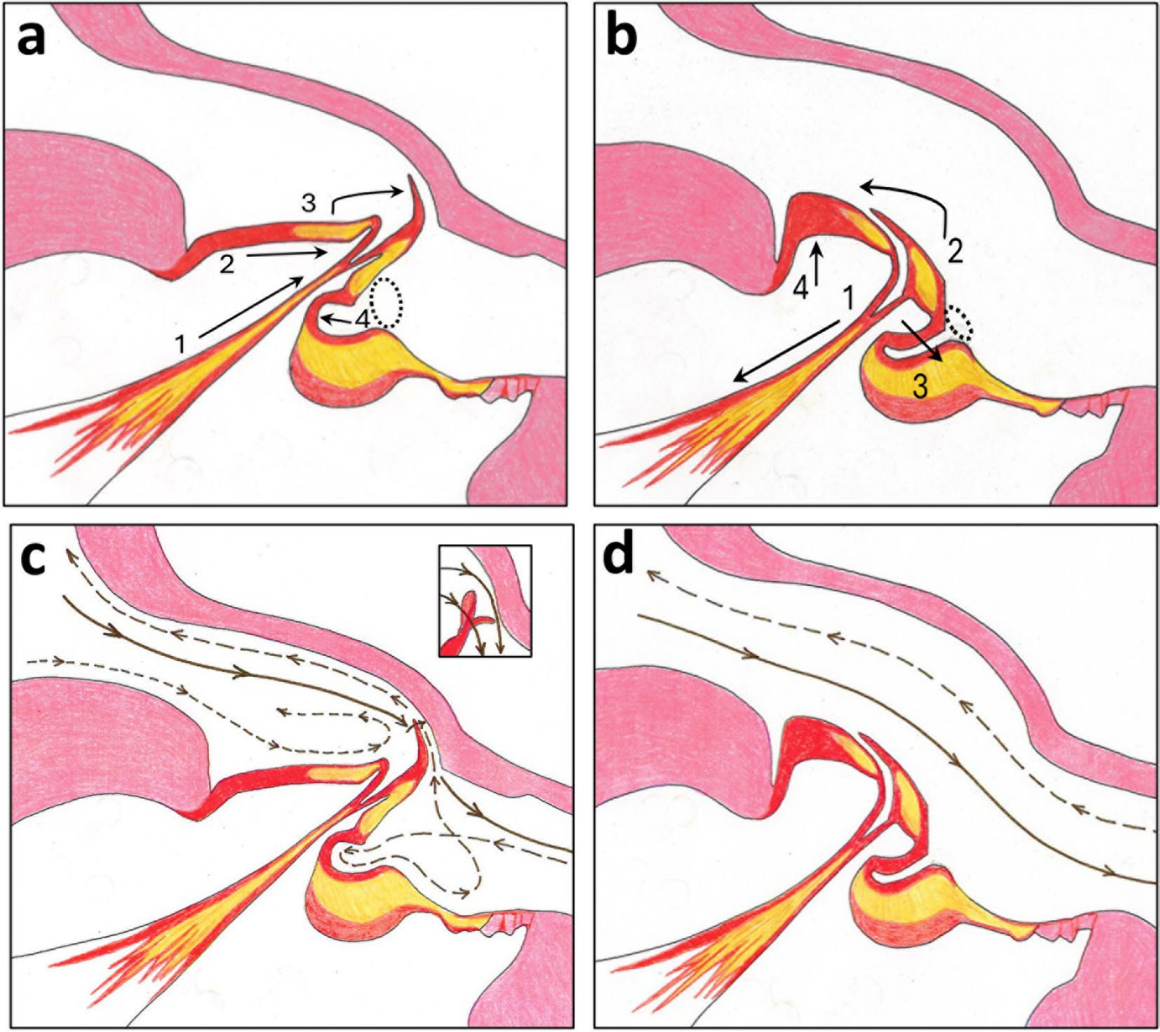
Interpretive drawings explaining the new model for opening and closing mechanisms of the sharpshooter precibarial valve and fluid flow through it. Cartoons show cuticular structures only. (a) Saggital section of the valve when closed and the valve muscle is relaxed showing movements performed to close the valve; based on a tracing of Fig. 2a. (b) Saggital section of the hypothesized appearance of the opened valve showing movement performed to open the valve. Numbers and arrows represent order of movements described in text. (c) Diagram of fluid flow through the precibarium when the valve is being opened or closed. (d) Diagram of fluid flow through the precibarium when the valve is open. For c and d: short-dash line, outward fluid movement for swallowing; long-dash line, fluid moving in/being taken up for later ingestion; solid line, fluid moving out/egestion (for c, rinsing egestion; for d, discharging egestion). For color version: colors are based on those in Fig. 2, that is, red, resilin; yellow, sclerotin; pink; procuticle. For black-and-white version: dark gray, resilin; pale gray, sclerotin; medium gray, procuticle.

 A diagram of our hypothesized operational mechanism for closing and opening of the sharpshooter precibarial valve is shown in Fig. 6. These drawings are not schematic like the published *P. spumarius* model^12^but instead drawn to be as close to anatomically correct as possible.

Closure of the precibarial valve can be directly ascertained from the LM sections of *G. atropunctata* and *H. vitripennis* in Results. Figure 6a shows a diagrammatic trace of the cuticular anatomy from sharpshooter micrographs. Probably based on complete cold-anesthetization of the dissected insects, the sectioned valve was fully open and relaxed in this study’s images. We agree with the authors of the *P. spumarius* model^12^ that closure would not require action of an antagonistic muscle. We also agree with the latter authors that backwash of fluid from the closure of the cibarial diaphragm could pick up the valve and facilitate closure (first hypothesized as a “pressure-sensitive check valve” function^20^. However, we disagree with the *P. spumarius* model^12^ that such fluid flow would be the sole means of closure. Instead, we propose that release of cuticular elasticity combined with relaxation of the valve muscle also contributes to closing the valve.

Events numbered in Fig. 6a unfold sequentially but near-simultaneously as follows. (1) As the precibarial valve muscle relaxes and extends, elastic tension in the lefthand basin cuticle (built up when the cuticle is compressed and bulging during opening, further described below) is released. (2) This release pushes the sclerotized 1/3 of the basin cuticle towards the right, which collapses the tendon tips together to adjoin, which then apply pressure to the stiff, sclerotized portion of the valve. This release of elastic energy causes (3) the valve to rotate clockwise from the open state (see below). As the valve rotates upward/rightward, (4) the highly elastic joiner cuticle unfolds and puts pressure on the stable cuticular pad leftward (in the image), causing the flaccid valve muscle and tendon to buckle slightly. In turn, the stiffness of the cuticular pad causes it to remain in place, forcing the entire basin and valve assembly to bulge upward. This causes the *bell-like invagination* (*Bli*) to open, and its pit/ring (*Ring*) opening (dashed line) to rotate and open facing to the right in the image. The resulting height between the basin height/length of the closed valve, and the height/depth of the bell-like invagination (*Bli*) relative to the “lower” distal enclosure gives the precibarium a step-like aspect (when viewed from the side in Fig. 6a, or in views from the lumen in SEM images, Fig. 4a).

### Hypothesized model for operational mechanism: Opening the valve

 We have no direct views via sagittal LM sections of the appearance of an open valve. However, we have extensive SEM images of the epipharyngeal half of the precibarium of *G. atropunctata* (only 5 out of 40 insects examined^16^ are shown herein), especially differences in appearance when the valve is closed versus open. These SEM images strongly support the hypothesized sharpshooter operational mechanism. In addition, SEM views of the epipharyngeal half of the precibarium in *P. spumarius* support that the sharpshooter mechanism is plausible for that species as well.

To explain the sharpshooter operational mechanism, events numbered in Fig. 6b again unfold sequentially but near-simultaneously, as follows. (1) The opening mechanism begins when the precibarial valve muscle contracts and shortens. The tendon pulls the basin and valve assembly downwards (in the image), causing (2) the elastic 2/3 of the basin cuticle (left side of the basin) to strongly bulge upward because the thick procuticle to the left of the basin (in the image) holds the basin cuticle in place. (3) The bulging causes the two tendon tips to unfold, lengthen and splay apart, rotating the valve counter- (anti-)clockwise until it fits into the triangular groove in the basin that houses it. Because the cuticular pad remains stationary, the elastic joiner cuticle buckles inward and constricts the entrance to the invagination (*Bli*). In the sharpshooter model, the rotation/opening of the valve reduces the step-like appearance (from upper view in the lumen), allowing the precibarium to take on the appearance of a slanted tube, which would reduce obstruction of inward fluid flow.

The vision for this closure mechanism in our work is different from the seeming-closure in the *P. spumarius* model shown in **Fig. S4** (Supplemental material) (from Fig. 3 in^12^. We hypothesize that the *P. spumarius* model was developed in part due to an artifactual appearance of the *P. spumarius* valve in Fig. 5c. It is likely that the entire valve in the latter image was pushed downward due to buckling of the section. Thus, the precibarial valve of sharpshooters is hinged, not a solid, flat extension of cuticle envisioned in the *P. spumarius* model^12^ (Supplementary **Fig. S3**); it does not move up and down in the lumen of the precibarium, but radially on its hinge like the hands of a clock, as originally suggested in^5^ (see Figs. 13.7 A, B, C therein).

### Implications of the sharpshooter operational model for fluid flow

 As mentioned in the Introduction, there are several aspects of the *P. spumarius* operational model^12^ that are accepted herein as accurate. Notably, present findings concur that the sharpshooter valve is closed when the precibarial valve muscle is relaxed. Yet, the most important and definitive finding herein is that the precibarial valve is *directly* attached to the precibarial muscle via its tendon and therefore is *hinged*, causing direct movement of the valve in a rotating fashion like the hands of a clock. A hinged valve can move rapidly in an open-close-open-close fashion, termed “fluttering” and originally diagrammed in Figs. 13.7 A, B, C in^5^. Also very important is the new finding herein of *valve tip flapping*. When combined with hinged movement of the valve, flapping would produce a distinctive, high-frequency fluttering of the valve, matching high-frequency EPG waveforms that detect in-out directionality of biopotentials caused by fluid flow^5–7^. The findings herein make such valve fluttering highly likely, and probably capable of propelling small amounts of fluid outward from the distal enclosure area, by themselves.

The existence of egestion (outward fluid flow from the precibarium through the food canal and out the stylet tips) has been definitively supported and hydrodynamically studied for sharpshooters^9–11,13,30^. Two types of egestion have been proposed for these insects^5,22^: (1) *discharging egestion*, in which the full contents of the nearly-filled cibarium are rapidly discharged out the stylet tips (also termed “spitting), and (2) *rinsing egestion*, in which only the smaller volume held in the precibarium (especially distal to the valve) is ejected (also termed “dribbling”).

To understand discharging egestion/spitting, first we need to understand ingestion. The cibarial dilators are lifted and the precibarial valve is held open, taking up fluid into the cibarium until it is filled (Fig. 6d, dashed line). During swallowing (ingestion), steady, unequal falling of the cibarial diaphragm (Supplementary **Fig. S2b**) forces cibarial fluid towards the true mouth. The start of swallowing pushes fluid distally into the precibarium and under a partially open precibarial valve, aiding closure of the valve (Fig. 6c, short-dashed line), while the bulk of fluid is then swallowed proximally past the true mouth into the pharynx (mistakenly identified as the *mesenteron* in Supplementary **Fig. S3**^12^). This is the major mechanism of sustained ingestion. Thus, findings from the present study confirm that the valve functions as a pressure-sensitive check valve to facilitate valve closure and swallowing, as first proposed in^34^. The mechanism of discharging egestion/spitting is rapid, complete and equal dropping of the cibarial diaphragm powered by cuticular elasticity (Supplementary **Fig. S2a**), *while the precibarial valve is actively held open by muscular action*, expelling most or all of the cibarial contents through the precibarium and out the stylets (Fig. 6d)^10,22,31^.

The mechanism of rinsing egestion/dribbling was previously thought due exclusively to valve fluttering, a mechanism still supported by the present work. However, an additional mechanism is now supported. After rapid release and fall of the cibarial diaphragm facilitating closure of the precibarial valve (Fig. 6c, short-dashed line), the space on the sides of the valve flap will likely allow some fluid to pass even a closed valve. In both swallowing and egestion, when the valve is closed, small amounts of fluid will continuously be squeezed past the leaky valve flap into the distal enclosure and forced outward, resulting in rinsing egestion/dribbling for every swallow the insect takes (Fig. 6c, solid line). It is likely that both mechanisms of rinsing egestion/dribbling could occur at the same time. Continuous rinsing egestion/dribbling washes out the distal enclosure and its D-chemosensilla. This would explain the “clean” state of the distal enclosure seen in most SEM images^9,22^. Accordingly, the likely primary function of continuous rinsing egestion is to keep the D-sensilla clean and clear, to support their vital sensory function in feeding^20^.

In a previously unrecognized mechanism, small amounts of cibarial suction could cause uptake of a small amount of fluid into the precibarium, both into the distal enclosure and into the basin via the leaky valve (Fig. 6c, long-dash line). Fluid would mix around between the basin and distal enclosure, including into the *Bli*, due to vortices formed^10^but eventually would pass through the leaky valve into the cibarium where it will be swallowed past the true mouth (be ingested). Such micro-ingestion (or “sipping”)^5^ is a second explanation for the trail of microbial slime seen moving through the partially closed valve of *H. vitripennis* in Fig. 3d, e, and f. Sequential sipping-and-dribbling could be an additive mechanism for rinsing egestion, thus further supporting the existence of rinsing egestion via structural anatomy of the valve.

Alternating sipping and dribbling could be the mechanism for two-stage chemical testing of fluid proposed upon initial discovery of the leafhopper precibarial valve^20^. A small amount of fluid could be sipped for first-stage testing by the gustatory D-sensilla in the distal enclosure. If compounds are acceptable, the valve could be opened for second-stage testing by the P-sensilla. Only after acceptance by both sets of sensilla would swallowing ensue. This idea is supported by the finding that ablation of the nerve from only the D-sensilla completely abolishes discrimination of compounds involved in host acceptance and finding of xylem^34^.

### **Implications for inoculation of*Xylella fastidiosa***

 Findings presented herein provide concrete anatomical evidence for both previously hypothesized types of egestion, that is, discharging egestion/spitting and rinsing egestion/dribbling^22^. In so doing, we support *Xf* acquisition and inoculation mechanisms previously hypothesized but not or only partially demonstrated^6,7^. Together, the present findings definitively support four important ways the recently published “vector regulation” hypothesis^16^ can occur. In this hypothesis, the insect’s fluid movements in the functional foregut can contrain and even regulate *Xf* biofilm development by “flushing out” bacteria to be egested into the plant^5,7^.

First, as described above, the finding that the precibarial valve is hinged and directly moved by the valve muscle strongly supports that both or either valve fluttering and/or leaking can lead to rinsing egestion/dribbling, which can carry microbes along thus providing a mechanism of *Xf* inoculation^5,22^. Valve fluttering is also hypothesized as the biological mechanism of an electropenetrography (EPG) waveform associated with *Xf* inoculation^5,7^. *Xf* bacteria present especially in the distal enclosure would be rinsed out. Thus, while discharging egestion may be most epidemiologically important for *Xf* inoculation, rinsing egestion probably also contributes^5^.

Second, this work directly visualizes for the first time that particles (putatively microbes) can be suspended in fluid within the distal enclosure when the valve is closed. If the particles observed in the present study are microbes, their type and source is not known because the *H. vitripennis* sectioned were field-collected then maintained on clean plants not infected with *Xf*. A previous SEM found highly diverse morphologies of microbes in the precibarium of field-collected *H. vitripennis*; these did not resemble the typical “shag carpet” appearance of a monoculture of *Xf*^22^. Nonetheless, the present imaged suspension of particles, likely microbes, generally supports the “flying syringe” hypothesis for *Xf* acquisition. That is, that at least for the first few minutes after uptake of any microbes (including but not exclusively *Xf*) they can be suspended (or re-suspended after initial adhesion) in a fluid column in the functional foregut prior to egestion or adhesion^22^. Third (and related to the second implication), the triangular shape of the presumed microbial biofilm and trail past the leaky valve shows that biofilm shape and position can be altered by the behavior of the insect.

A fourth implication for the vector regulation hypothesis from the present findings is that the pit/ring (*Ring*) becomes a narrow slit when the valve is fully open, thus the *bell-like invagination* (*Bli*) becomes a nearly-closed pocket. Any microbes held therein could be trapped. Repeated cycles of sipping or swallowing alternating with rinsing egestion would likely push the microbes out of the invagination and thence the stylet tips; however, perhaps some microbes could be retained in the invagination if they adhere strongly to cuticle, providing a “refuge” for bacteria to re-colonize the foregut after cleaning-out by the insect^16^.

The mechanism of precibarial valve operations during both types of egestion can aid development of pest management tactics in several ways. First, identification of EPG waveforms that represent valve fluttering and their correlation with *Xf* inoculation^7^ explains quantitative EPG findings that grapevine varieties can be resistant to the same vector inoculation behaviors^35^allowing for future pyramiding of novel resistance traits into resistant accessions. Second, anti-microbial peptides or other compounds in the plant could be genetically or otherwise manipulated to increase concentration in xylem, so that the when the sharpshooter ingests/egests a high enough concentration of the compound it could to kill the *Xf* (or at least reduce titer) found in the precibarium, even in the refuge of the precibarial invagination.

Accordingly, the findings herein support the long-held view that understanding the functional anatomy of the precibarial valve is one of the most important keys to unlocking the mysteries of *Xf* inoculation and improving pest management. Given the strong similarities in appearance between SEM views of precibarial structures in spittlebugs and sharpshooters, we agree with the *P. spumarius* study authors^12^ that the internal structures and mechanism of valve operation are likely to be similar between the two taxa. We therefore propose that our sharpshooter valve operational model is more similar to spittlebug valve operation than the model previously proposed^12^but only a re-analysis of *P. spumarius* structures with methods similar to ours could test that conclusion.

## Supplementary Information

Below is the link to the electronic supplementary material.

Supplementary Material 1

## Data Availability

All information generated or analyzed during this study are included in this published article.
