# Supplementary material for: Functional anatomy of the sharpshooter precibarial valve supports its role in probing behaviors that control inoculation of Xylella fastidiosa
Source: Sci Rep. 2025 Sep 30;15:34078. doi: 10.1038/s41598-025-14208-4 (PMC12484689; doi:10.1038/s41598-025-14208-4)
Supplement: Supplementary file 1 — Supplementary Material 1 [file 41598_2025_14208_MOESM1_ESM.docx]

**SUPPLEMENTAL INFORMATION FOR**

**Functional anatomy of the sharpshooter precibarial valve definitively reveals**

**its role in probing behaviors that control inoculation of *Xylella fastidiosa***

Elaine A. Backus and Damien Laudier

For the benefit of the reader, we reproduce herein some of the most important figures and original captions from previously published work on the auchenorrhynchan precibarial valve (**Figs. S1, S3 and S4**). Also, the positions of the cibarial diaphragm and muscles for the insects in **Fig. 2** in the main paper are included (**Fig. S2**).

**Figure S1.** **Earliest published^1^ view of the precibarial valve muscle in sharpshooters.** Side view of the interior of the precibarium of the green sharpshooter, *Draeculacephala minerva* Ball (subfamily Cicadellinae). *(The upper plate is the hypopharynx and the lower is the epipharynx – added by EAB.)* The P-sensilla on the left side of the insect are visible in the basin (arrow), with the nerve attached (Pn). While the precibarial valve is hidden from view, its muscle (mu) and the muscle attachment to the wall of the clypellus (clpl; also known as the anteclypeus) is visible. X360. Scale indicates 20 um. Figure and caption from^1^ with additions for the present paper in italics; re-printed with permission of the publisher via Copyright Central. To conform to format for the rest of the figures (and those in^2^), this figure was flipped around the vertical axis using Photoshop.

**Figure S2.**  **Positions of the cibarial diaphragm in sharpshooters used in Fig. 2 in the main article.**  a) The cibarial diaphragm is collapsed tightly against the cibarial floor in *G. atropunctata*, thus fluid was still or had just finished flowing outward, for **Figs. 2a, b, and c** in the main article. b) The cibarial diaphragm is elevated with space above the cuticular floor of the cibarium in *H. vitripennis*, although the diaphragm cuticle was broken during sectioning. Unequal elevation denotes that the diaphragm was probably dropping during swallowing, thus fluid flowed both towards the valve in the precibarium and towards the true mouth (*****), for **Figs. 2d, e, and f** in the main article.

*****

*****


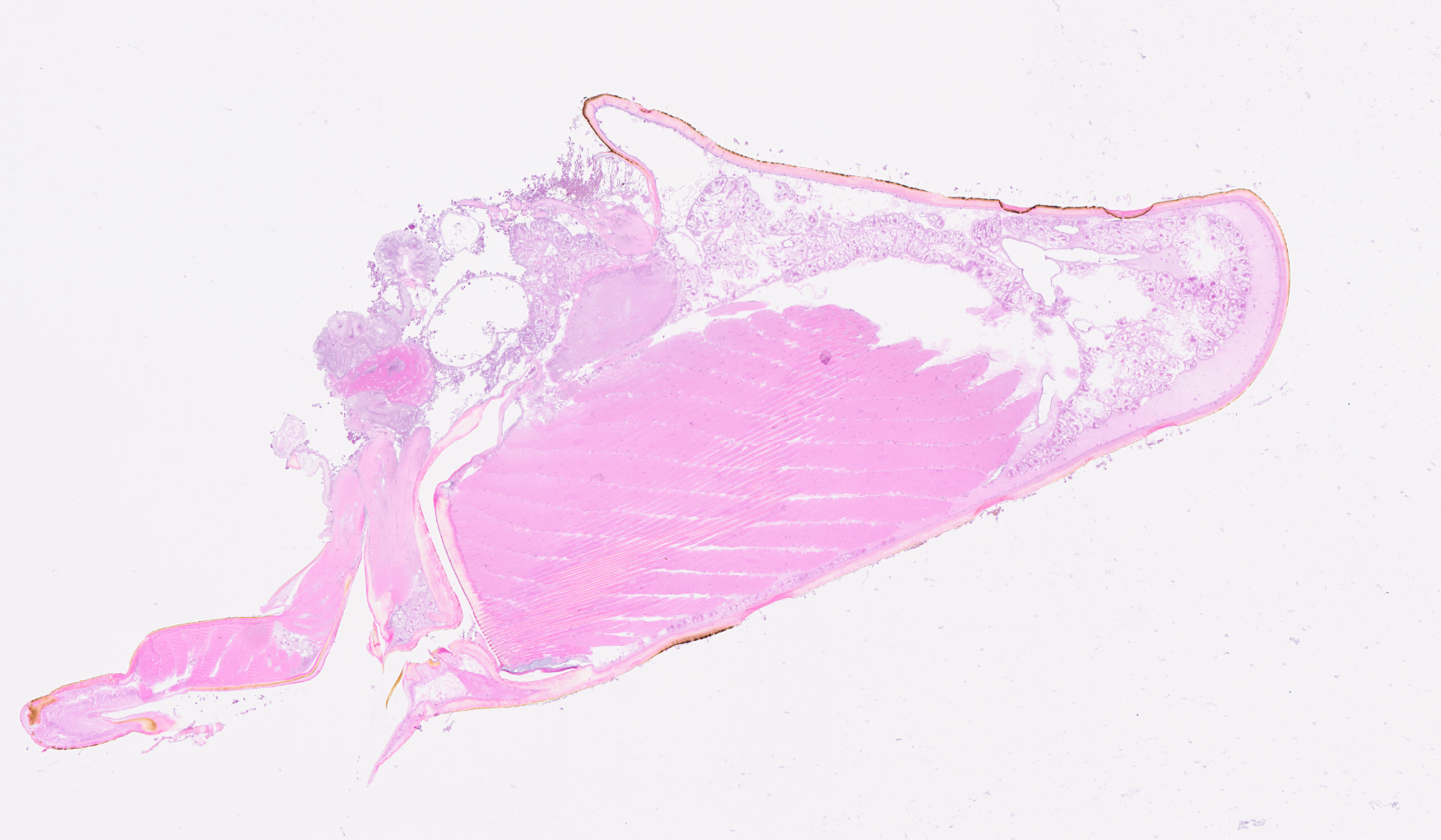

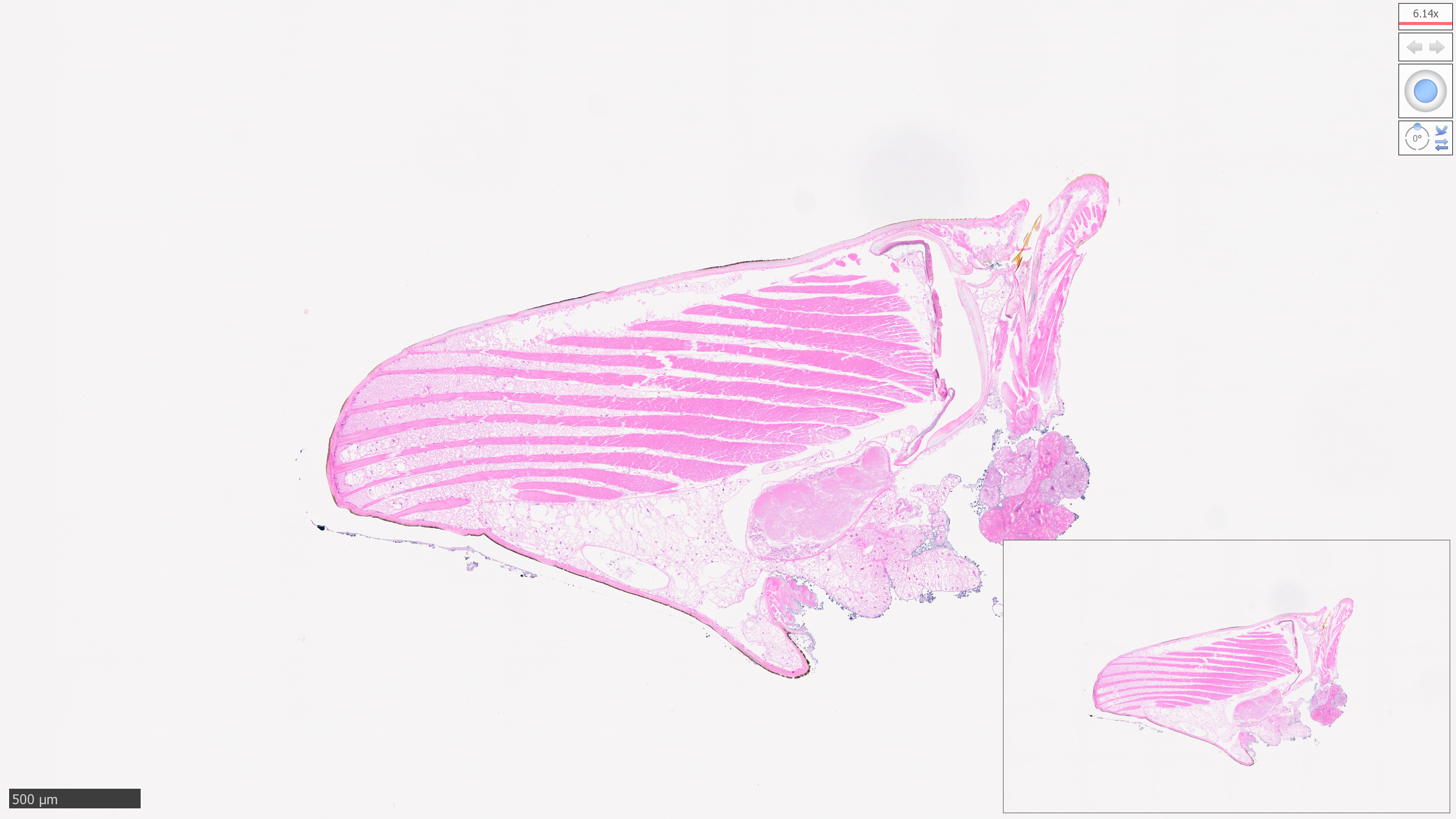


**a**

**b**

Note the differential uplift of the diaphragm in *H. vitripennis* (**Fig. S2b**) with the left side more uplifted than the right side. This occurs when the diaphragm lowers asymmetrically to push fluid towards the true mouth (asterisk)^3^. When withdrawing to pull fluid inwards, the diaphragm pulls symmetrically; when egesting, the diaphragm is thought to drop symmetrically^4^. Therefore, this insect was actually swallowing (diaphragm dropping asymmetrically) when it died. Therefore, the triangular microbial biofilm and trail in the precibarium appearing to move inwards may reflect prior uptake of fluid before the valve was closed. It is likely that the velocity/power of inward flow while the valve is open (uptake for ingestion) would be stronger than outward flow while the valve is closed. This higher velocity of inward flow could account for the inward-bending shape of the microbial biofilm and trail.

**Fig. S3. Previously published^2^ images of *P. spumarius* precibarium in attempt to identify the attachment of the muscle to the valve. (**D) LM cross-section taken below the level of the ring, the cuticle forming the bell-like invagination (Bli) and the glandular epithelium are visible. (E) LM cross section at the level of the basin-like structure in which is visible part of the muscle (Blm). Scale bar = 20 µm. Figure and caption from^2^ with addition of white “lumen” and arrow in part D by E. A. Backus; re-printed with permission of the publisher via Copyright Central.

lumen

While the original authors interpret **Fig. S3E** as showing the *Basin-like muscle* (*Blm*) (a.k.a. the precibarial valve muscle), the image is very fuzzy and more likely to be cuticular because it is confluent with and resembles the cuticle at the edge of the fan-shaped opening.

In addition, the fan-shaped opening in **Fig. S3E** is the lumen of the precibarium itself. This is because the lumen in part D is clearly shown lying next to the cross-sectioned, thick cuticle of the hypopharynx (plate on the right), across from the thinner-cuticle epipharynx on the left. The same cuticle border is seen to the right of the fan-shaped opening in part E. Therefore, the cuticular ridge identified as Blm attaches to the edge of the lumen. Given the stated depth of the section, it may be a slice through the valve tendon or the joiner cuticle, but it is very difficult to independently ascertain its identification without a clear view of the valve to which the tendon must be attached.

**Figure S4. Previously proposed^2^ operational mechanisms of the precibarial valve in *Phylaenus spumarius*** (A) Schematic drawing of the head and mouth parts of *Philaenus spumarius* showing the rostrum (Ros) (*also known as the labium – EAB*), the stylet food canal (Sty), the hypopharynx (Hyp) and the epipharynx (Epi), the cibarium with its muscle (Cbm) and diaphragm (Cbd) and the precibarial valve in which is the depicted the bell-like invagination (Bli), the flap (Flp) and the basin-like muscle (Blm). The dashed square represents the precibarial area below schematized at the three different steps during fluid uptake: at the relaxed state with the valve closed (first step); during feeding (*in the narrow sense of ingestion; Backus 2000 – more precisely known as fluid uptake – added by EAB*) with the valve opened (second step); when the cibarium chamber is filled and the valve is closed (third step). (B) First step: relaxed state, the valve is closed. The flap (Flp) lays aligned to the hypopharynx (Hip), the muscles (Cbm, Blm) are relaxed. (C) Second step: the insect starts feeding and the valve opens. The cibarial muscle (Cbm) contracts (solid lines), pulling the food pump open. The basin-like muscle (Blm) contracts (solid lines), bringing along the flexible cuticle of the basic-like structure (Bls), the ring (Rin) bends towards the cibarium, pivoting on the base attached to the distal surface of the lumen and the flap (Flp) is lowered and leaned toward the basin-like structure (Bls). that time, the sap is free to pass by (dashed line); (D) Third step: The cibarium chamber is filled up, the valve closes. Shortly before the downward strokes of the cibarial diaphragm (Cbd), the basin-line muscle (Blm) relaxes (dotted lines), the flexible cuticle of the basin-like structure (Bls) returns to its original position. Hence, the flap (Flp) moves up and goes towards the hypopharynx (Hyp) and the ring (Rin) tends to turn back to its invagination (Bli), filling it up and creating a pressure that pushes the flap (Flp) against the hypopharynx (Hyp). When the precibarial valve is closed, the cibarium pumps the sap towards the mesenteron (Mes) (*This is incorrectly identified; the mesenteron is the midgut, but the portion of the alimentary canal following the true mouth is the foregut, divided into the pharynx then esophagus^3^ – added by EAB*), and part of it is pushed towards the precibarial valve. The pressure of the fluid (dashed line) pushes it into the internal wall of the flap (Flp), forcing it to be “attached” (*actually pressed against – EAB*) the hypopharynx (Hyp). Figure and caption from^2^ with additions in italics for the present paper by E. A. Backus; re-printed with permission of the publisher via Copyright Central.

**References**

1 Backus, E. A. & McLean, D. L. The sensory systems and feeding behavior of leafhoppers. II. A comparison of the sensillar morphologies of several species (Homoptera: Cicadellidae). *J. Morphol.* **176**, 3-14 (1983).

2 Ruschioni, S. *et al.* Functional anatomy of the precibarial valve in Philaenus spumarius (L.). *PLoS ONE* **14**, doi:10.1371/journal.pone.0213318 (2019).

3 Snodgrass, R. E. *Principles of Insect Morphology*. (McGraw-Hill Book Co., 1935).
